# Synthesis and Preliminary Evaluation of an ASGPr-Targeted Polycationic *β*-Cyclodextrin Carrier for Nucleosides and Nucleotides

**DOI:** 10.3390/pharmaceutics16030323

**Published:** 2024-02-26

**Authors:** Jang-Ha Ryu, Weizhong Zheng, Xiao-Hong Yang, Hassan Elsaidi, Jim Diakur, Leonard I. Wiebe

**Affiliations:** 1 Independent Researcher, Hwasung-city 18245, Republic of Korea; jryued@hotmail.com; 2 Independent Researcher, Edmonton, AB T6W 1T4, Canada; zhengweizhong@hotmail.com; 3 Independent Researcher, Edmonton, AB T6G 1Z2, Canada; xyang@ualberta.ca; 4 Department of Pharmaceutical Chemistry, Faculty of Pharmacy, University of Alexandria, El Sultan Hussein St. Azarita, Alexandria 21521, Egypt; elsaidi@ualberta.ca; 5 Independent Researcher, Winnipeg, MB R3T 0P4, Canada; jdiakur@mymts.net; 6 Faculty of Pharmacy and Pharmaceutical Sciences, 2-35 Medical Sciences Building, University of Alberta, 8613-114 St., Edmonton, AB T6G 2H7, Canada

**Keywords:** antiviral nucleosides, arabinosyl adenine (araA, vidarabine), arabinosyl adenine 5′-monophosphate (araAMP), asialoglycoprotein receptor (ASGPr), beta-cyclodextrin (CyD), endocytic receptors, HepAD38 cells, molecular docking, nucleotide delivery, polycationic cyclodextrins, targeted drug delivery

## Abstract

Most antiviral and anticancer nucleosides are prodrugs that require stepwise phosphorylation to their triphosphate nucleotide form for biological activity. Monophosphorylation may be rate-limiting, and the nucleotides may be unstable and poorly internalized by target cells. Effective targeting and delivery systems for nucleoside drugs, including oligonucleotides used in molecular therapeutics, could augment their efficacy. The development of a carrier designed to effect selective transmembrane internalization of nucleotides via the asialoglycoprotein receptor (ASGPr) is now reported. In this work, the polycationic, polygalactosyl drug delivery carrier heptakis[6-amino-6-deoxy-2-O-(3-(1-thio-β-D-galactopyranosyl)-propyl)]-β-cyclodextrin hepta-acetate salt (GCyDAc), potentially a bifunctional carrier of (poly)nucleotides, was modeled by molecular docking in silico as an ASGPr-ligand, then synthesized for testing. The antivirals arabinosyl adenine (araA, vidarabine, an early generation antiviral nucleoside), arabinosyl adenine 5′-monophosphate (araAMP), and 12-*mer*-araAMP (p-araAMP) were selected for individual formulation with GCyDAc to develop this concept. Experimentally, beta cyclodextrin was decorated with seven protonated amino substituents on the primary face, and seven thiogalactose residues on its secondary face. AraA, araAMP, and p-araAMP were individually complexed with GCyDAc and complex formation for each drug was confirmed by differential scanning calorimetry (DSC). Finally, the free drugs and their GCyDAc complexes were evaluated for antiviral activity using ASGPr-expressing HepAD38 cells in cell culture. In this model, araA, araAMP, and p-araAMP showed relative antiviral potencies of 1.0, 1.1, and 1.2, respectively. In comparison, GCyDAc-complexes of araA, araAMP, and p-araAMP were 2.5, 1.3, and 1.2 times more effective than non-complexed araA in suppressing viral DNA production. The antiviral potencies of these complexes were minimally supportive of the hypothesis that ASGPr-targeted, CyD-based charge-association complexation of nucleosides and nucleotides could effectively enhance antiviral efficacy. GCyDAc was non-toxic to mammalian cells in cell culture, as determined using the MTS proliferation assay.

## 1. Introduction

Many antiviral and anticancer nucleosides, like arabinosyl adenine (araA; vidarabine), are prodrugs. Their antiviral activity requires sequential molecular bioactivation to their respective 5′-mono-, 5′-di-, and 5′-tri-phosphate nucleotide anabolites [1,2]. AraA is an early generation antiviral and anticancer therapeutic nucleoside [3] that is no longer in clinical use because it has been superseded by more specifically targeted and more potent drugs [4]. Conversion of araA to araA-triphosphate (araATP) is a typical, essential metabolic elaboration [5] that does not require viral enzymes at any of the three phosphorylation steps for its antiviral action. AraATP competitively inhibits DNA polymerases, thereby preventing DNA elongation when incorporated into terminal positions of both host and viral DNA. Because viral DNA synthesis is sensitive to lower doses of the drug, there is a selective antiviral effect, such that araATP is up to 40 times more toxic to some DNA viruses than to their host cells, although large doses of araA are also cytotoxic to dividing host cells [6,7,8,9].

Passage of araA across cell membranes is promoted by at least three transporter proteins, classified as nitrobenzylthioinosine sensitive (*es*), nitrobenzylthioinosine insensitive (*ei*), and concentrative (N1) [10,11]. AraA has a low aqueous solubility and susceptibility to metabolic degradation in vivo [12], together with its reported rate-limiting intracellular monophosphorylation [13]. Historically, this provided justification for the pursuit of therapy using araAMP rather than araA [14,15], because araAMP is inactivated relatively slowly in humans, is highly water soluble, and may therefore be a good substrate for vesicular nucleotide transport [16]. Unfortunately, most monophosphates (e.g., araAMP) are not metabolically stable in vivo. Monophosphate nucleotides in general cannot diffuse across cellular membranes because of their double negative charge at physiological pH [17,18], although nucleotides may be transported by nucleotide transporters [19]. Poly/oligo-nucleotide transport proteins appear not to exist in mammalian systems, but the use of carriers, especially those exploiting vesicular transmembrane transport, represents an important approach to promote delivery of poly/oligo-nucleotides into target cells [16,20,21].

Cyclodextrins (CyDs; cyclic α-(1,4) linked glucose oligosaccharides) are non-toxic, slowly metabolized ‘molecular buckets’ with wide-end internal cavity diameters of 4.9, 6.2, and 7.9 Å for α-, β-, and γ-CyDs, respectively. These dimensions are ideal for complexing low molecular weight lipophilic drugs (guests), forming inclusion complexes that alter the solubility, in vivo stability, toxicity, and bioavailability of the inclusion guest. The stability of guest/host inclusion complexes is governed by several association/dissociation principles that can be selectively modified via chemical manipulation to modulate drug release parameters. The literature is replete with discussions and reviews of the principles and challenges of engineering carriers that pose specific demands [22,23,24].

Research into nucleoside delivery using naturally occurring cyclodextrins was first reported in 1970 [25]. A detailed study of araA complexes formed with β-cyclodextrin (β-CyD) and hydroxypropyl-β-CD was reported in 1990 [26], but to our knowledge, the clinical application of CyDs as nucleoside delivery devices has not been extended to the delivery of partially activated nucleoside phosphates (e.g., monophosphates). Polynucleotide delivery is particularly important because of the therapeutic potential of antisense, mRNA, and other oligonucleotide therapeutics. Common approaches to oligonucleotide delivery involve chemical modification to mimic other drugs, covalent conjugation to cell-targeting and/or cell-penetrating moieties, nanoparticle formulation with endogenous vesicles, spherical nucleic acids, DNA cages, and smart materials [27,28]. Challenges to the development of effective and targeted carriers for nucleotides in general are related in part to the metabolic and transport complexities of nucleosides and nucleotides [29].

The utilization of charged CyDs for delivery of oppositely charged araA nucleosides has been proposed as a medium for araAMP delivery into target cells [30]. Earlier modeling of mononucleotide binding has demonstrated the impact of a single oppositely charged (protonated amino) group on either the primary or secondary face of β-CyD [31] and on per-aminated β-CyD [32].

**Figure 1:**
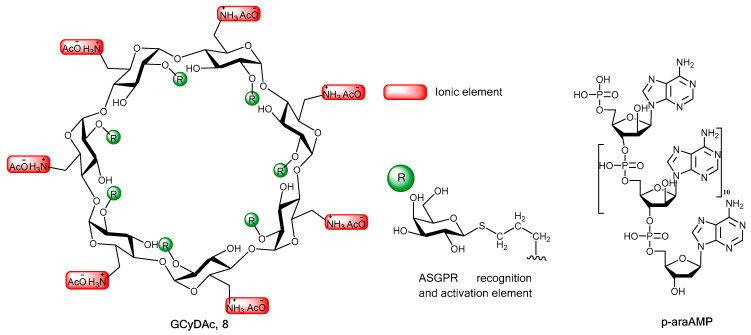
Schematic representations of GCyDAc (**8**; left) and p-araAMP (right), the synthetic 12-*mer* oligonucleotide deoxyadenylyl-(5′-3′)-(arabinoadenylic)_10_-5′-arabinoadenosine monophosphate. The ionic substituents are on the primary face and the 1(thio-β-D-galactopyranosyl)propyl-ASGPr binding elements (R, center) are on the secondary face of the rigid 7-glucose toroidal CyD backbone.

The current manuscript extends the concept of charge-association between nucleotides and cationic β-CyDs to include targeting the asialoglycoprotein receptor (ASGPr) for site-specific uptake of the drug/host complex. The synthesis of GCyDAc (**8**, Figure 1), targeted to ASGPr for cellular delivery of antiviral (poly)nucleotide-CyD complexes to hepatocytes, is now reported. The influence of complexation of **8** with araA, araAMP, and p-araAMP on their anti-HBV potencies was estimated using a mammalian hepatocyte model [33,34]. Cytotoxicity of GCyDAc to mammalian cells was determined by assay using a commercial MTS assay.

## 2. Chemistry, Formulation and Molecular Docking

β-Cyclodextrin (β-CyD), arabinosyl adenine (araA), and arabinosyl adenine monophosphate (araAMP), purchased from Sigma-Aldrich Canada, (Oakville, ON, Canada), were dried over P_2_O_5_ at room temperature for 24–48 h before use. Other chemicals and solvents were purchased from Sigma-Aldrich Co. and Fisher Scientific Company (Ottawa, ON, Canada). Solvents were dried according to literature procedures. The solvents used for synthesis were reagent grade unless specified otherwise, and other chemicals and solvents were of analytical grade. The 12-*mer* oligonucleotide deoxyadenylyl-(5′-3′)-(arabinoadenylic)_10_-(5′-3′)-arabinoadenosine monophosphate (p-araAMP; Figure 1) was used as provided under contract by Nucleic Acid Protein Service (UBC, Vancouver, BC, Canada). Additional electrophoretic analyses of p-araAMP purity are described in the Supplemental Data file.

Thin-layer chromatography (TLC) was carried out on Kieselgel 60 F254 (Merck, Darmstadt, Germany) and visualization was accomplished by charring with 5% methanolic sulfuric acid. Column chromatography was performed using silica gel 60 (70-230 Mesh ASTM, 0.063–0.2 mm, Rose Scientific Ltd., Edmonton, Canada). A Labconco Freeze Dryer (Kansas City, MO, USA) was used for lyophilization. Differential scanning calorimetry (DSC) was performed on a DSC 120 (SEIKO SII, model SSC/5200) calorimeter. NMR spectra were recorded on either a Bruker AM-300 or a Varian VXR-500 spectrometer (300 and 500 MHz, respectively, for ^1^H; 75.4, and 125.74 MHz for ^13^C). Chemical shifts are quoted in ppm, referenced to residual CHCl_3_ at δ 7.27 for CDCl_3_ solutions or HOD at δ 4.82 (25 °C) for D_2_O solutions. Coupling constants (*J*) are reported in Hertz. ^13^C-NMR spectral assignments were aided by the J-MOD spin-echo technique in which methyl and methine carbon resonances appear as positive peaks, methylene and quaternary carbon resonances appear as negative peaks. MALDI mass spectra were obtained using an Applied BioSystems Voyager Elite/N_2_ laser instrument.

**Scheme 1:**
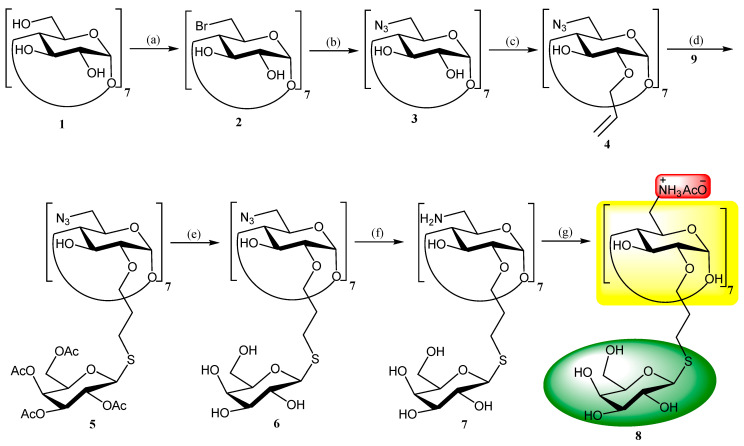
Chemical elaboration of β-CyD **1** to heptakis[6-amino-6-deoxy-2-O-(3-(1′-thio-β-D-galactopyranosyl)-propyl)]-β-cyclodextrin acetate **8** (GCyDAc). Reagents and conditions: (a) (C_6_H_5_)_3_P/Br_2_/DMF, 1 h; (b) NaN_3_, DMF, KI, 80 °C, 12 h, 60%; (c) NaH, AllylBr, DMF, 48 h, 40%; (d) **9**, AIBN, CH_3_CN, 70 ^º^C, 7 h, 61%; (e) NaOCH_3_- CH_3_OH, CH_3_OH, 30 h, 93%; (f) Ph_3_P/NH_4_OH, DMF, 48 h, 88%; (g) HOAc.

Commercially available **2** and **3** (Scheme 1) were prepared from **1** using literature procedures [35,36]. Briefly, heptakis(6-bromo-6-deoxy)-β-cyclodextrin **2** was prepared by direct halogenation of unprotected β-CyD **1** with (C_6_H_5_)_3_P/Br_2_/DMF; the ^13^C-NMR (75.5 MHz, D_2_O) spectrum of the crude product showed an upfield shift for C-6 to δ 34.4 ppm from δ 60.9 ppm for starting material (β-CyD). The crude bromide was subjected to a nucleophilic displacement reaction with azide to yield the corresponding heptakis(6-azido-6-deoxy)-β-CyD (90%) as a tan-colored solid. Due to the poor solubility of the crude azide, it was converted to fully acetylated compound upon treatment with acetic anhydride and pyridine to facilitate the column purification. The structure of the acetylated compound was confirmed with ^1^H-NMR and IR spectroscopy. Zemplen deacetylation yielded the pure azide, heptakis(6-azido-6-deoxy)-β-cyclodextrin **3**. Details are presented in the Supplementary Data file.

*Heptakis(6-azido-6-deoxy-2-O-allyl)-β-cyclodextrin* **4**: Sodium hydride powder (60%, 1.27 g, 31.63 mmol) was added to a solution of **3** (5.18 g, 3.95 mmol) in DMF (240 mL) under an atmosphere of argon at 0 °C. The reaction mixture was stirred at 0 °C for 1.5 h and then stirred overnight at room temperature. Allyl bromide (3.87 g, 32 mmol) was added dropwise, stirred at 0 °C for 1 h and then stirred overnight at room temperature. The solution was then cautiously poured into ice water (500 mL) and the precipitate was collected by filtration, then purified by column chromatography (hexane/EtOAc, 1:1) to afford **4** (2.56 g, 40%) as a white solid: ^1^H-NMR (CDCl_3_, 300 MHz): δ = 3.31 (t, 7H; H-2), 3.42–3.47 (dd, 7H; H-4), 3.52–3.59 (dd, 7H; H-5), 3.71–3.75 (m, 14H; H-6), 3.88–3.94 (t, 7H; H-3), 4.21–4.27 (dd, 7H; OC*H*_a_), 4.45–4.51 (dd, 7H; OC*H*_b_), 4.83 (d, 7H; H-1), 4.91 (s, 7H; OH), 5.24–5.35 (m, 14H; CH=C*H*_2_), 5.87–6.00 ppm (m, 7H; C*H*=CH_2_); ^13^C-NMR (CDCl_3_, 75 MHz): δ = 51.5 (C-6), 70.2, 73.0, 78.7, 84.7 (C-2, C-3, C-4, C-5), 73.6 (O*C*H_2_), 101.8 (C-1), 119.2 (*C*H_2_=CH), 134.3 ppm (CH_2_=*C*H); MS (MALDI) calcd for C_63_H_91_N_21_O_28_ *m*/*z* [M + Na]^+^ 1613.51, found 1613; elemental analysis calcd for C_63_H_91_N_21_O_28_ C, 47.57; H, 5.77; N, 18.49, found C, 47.65; H, 5.66; N, 18.19.

*Heptakis[6-azido-6-deoxy-2-O-(3-(2′,3′,4′,6′-tetra-O-acetyl-β-D-thiogalacto-pyranosyl)propyl)]-β-cyclodextrin* **5**: Cyclodextrin **4** (100 mg, 0.063 mmol) and **9** (280 mg, 0.77 mmol) were dissolved in CH_3_CN (40 mL). Azobisisobutyronitrile (AIBN; 30 mg, 0.18 mmol) was added under argon at 70 °C, and after stirring for 7 h, the solvent was removed under vacuum (water aspirator), and the residue was purified by column chromatograph (hexane/EtOAc/CH_3_OH, 5:5:1) to afford **5** (0.16 g, 0.0386 mmol; 61%) as a white foam: ^1^H-NMR (CDCl_3_, 500 MHz): δ = 1.87–1.94 (m, 14H; SCH_2_C*H*_2_), 1.97, 2.04, 2.06, 2.15 (4s, 84H; 28 × C*H*_3_CO), 2.58–2.87 (m, 14H; SC*H*_2_), 3.28 (t, 7H; H-2), 3.34–3.37 (m, 7H; H-4), 3.52–3.56 (m, 7H; H-6a), 3.67–3.84 (m, 28H), 3.95–4.17 (m, 28H), 4.52–4.58 (m, 7H; H-1′), 4.78 (s, 7H; O*H*), 4.90 (s, 7H; H-1), 5.06 (dd, 7H), 5.19 (m, 7H; H-2′), 5.43 (m, 7H; H-3′); ^13^C-NMR (CDCl_3_, 125 MHz): δ = 20.66, (28 × *C*H_3_CO), 27.12 (SCH_2_*C*H_2_), 29.98 (S*C*H_2_), 51.45, 60.37, 61.21, 67.20, 67.30, 68.0, 68.05, 70.35, 71.49, 71.78, 72.82, 74.25, 80.37, 84.50, 84.62, 101.65, 169.54, 170.02, 170.19, 170.30 ppm (28 × CH_3_*C*O); MS MALDI calcd for C_161_H_231_N_21_O_91_S_7_ *m*/*z* [M + Na]^+^ 4164.08, found 4164. Elemental anal calcd for C_161_H_231_N_21_O_91_S_7_: C, 46.70; H, 5.62; N, 7.10, found C, 46.44; H, 5.47; N, 6.96.

*Heptakis[6-azido-6-deoxy-2-O-(3-(1′-thio-β-D-galactopyranosyl)propyl)]-β-cyclodextrin* **6**: NaOCH_3_ (1 M, 18 mL) was added to a stirred solution of **5** (1.8 g, 435 mmol) in dry CH_3_OH (250 mL), and the reaction mixture was stirred for 30 h at RT. Water (30 mL) was added, and the reaction was stirred for 1 h to dissolve precipitates. The reaction was neutralized with Amberlite IR-120 H^+^ ion-exchange resin and filtered. The solution was concentrated under vacuum (water aspirator) and the resultant glassy residue was dissolved in H_2_O and freeze-dried to afford **6** as a white fluff (1.2 g, 93%). Selected NMR data,^1^H-NMR (D_2_O, 300 MHz): δ = 1.90–1.96 (m, 14H; C*H*_2_), 2.77–2.80 (m, 7H; SC*H*_2_), 4.47 (d, 7H; H-1′), 5.14 ppm (s, 7H; H-1); MS (MALDI) calcd for C_105_ H_175_ N_21_ O_63_ S_7_ *m*/*z* [M + Na]^+^ 2987.05.08, found 2987.

*Heptakis[6-amino-6-deoxy-2-O-(3-(1′-thio-β-D-galactopyranosyl)propyl)]-β-cyclodextrin* **7**: Azido CyD **6** (145 mg, 0.049 mmol) and triphenylphosphine (0.27g, 1.03 mmol) were dissolved in DMF (3 mL) and the mixture was stirred at RT for 30 min. Aqueous ammonia (3 mL) was added and stirring continued at RT for 48 h. The mixture was diluted with CH_3_OH and evaporated under vacuum. The residue was dissolved in H_2_O (40 mL) and washed with CH_2_Cl_2_ until no UV-active material was present in the organic layer. The aqueous layer was lyophilized to afford **7** (121 mg, 88%) as a solid. Selected NMR data, ^1^H-NMR (D_2_O, 300 MHz): δ = 1.84–1.93 (m, 14H; C*H*_2_), 2.68–3.10 (m, 14H; SC*H*_2_), 4.46 (d, 7H; H-1′), 5.19 ppm (s, 7H; H-1); ^13^C-NMR (D_2_O, 75 MHz): δ = 28.71, 28.76, 28.96, 43.52, 60.15, 60.9, 63.54, 70.37, 71.25, 71.62, 72.13, 72.68, 73.96, 74.7, 75.2, 76.44, 81.37, 82.17, 82.26, 82.29, 88.17, 88.3, 102.31 ppm; MS (MALDI) calcd for C_105_ H_189_ N7 O_63_ S_7_ *m*/*z* [M + Na]^+^ 2805.07, found 2804.

*Heptakis[6-amino-6-deoxy-2-O-(3-(1′-thio-β-D-galactopyranosyl)propyl)]-β-cyclodextrin acetate* **8** (GCyDAc). CyD **7** (86 mg, 0.031 mmol) was treated with aqueous HOAc (0.113 M, 1.92 mL), and the solution was lyophilized to give 84 mg of **8** as a solid. Selected NMR data, ^1^H-NMR (D_2_O, 300 MHz): δ = 1.84–2.14 (m, 35H; C*H*_3_CO and C*H*_2_), 2.68–2.91 (m, 14H; SC*H*_2_), 4.47 (d, 7H; H-1′), 5.27 ppm (s, 7H; H-1).

*2,3,4,6-Tetra-O-acetyl-1-thio-β-D-galactopyranose* **9** (O-Ac-TG). Thioacetamide (0.4 g, 5.4 mmol) was mixed with 2,3,4,6-tetra-*O*-acetyl-β-D-galactopyranosyl bromide (2.1 g, 5 mmol), and heated at 120 °C under argon for 10 min. The reaction mixture was allowed to cool to RT, then CH_3_OH (40 mL) was added to dissolve the solid. Solvent was removed under vacuum (water aspirator), and the residue was purified using column chromatography (hexane/EtOAc, 1:1) to afford the title compound **9** (1.33 g, 73%); ^1^H-NMR (CDCl_3_, 300 MHz): δ = 1.98, 2.04, 2.05, 2.09 (4s, 12H; 4Ac), 2.37 (d, 1H, *J* =10.1 Hz; SH), 3.92–3.97 (m, 1H; H-5), 4.03–4.17 (m, 1H; H-6a), 4.51–4.63 (m, 1H; H-6b), 4.54 (t, 1H; H-1), 4.99–5.03 (dd, 1H, *J* = 10.1; H-2), 5.15–5.30 (m, 2H; H-4 and H-3); ^13^C-NMR (CDCl_3_, 75 MHz): δ = 20.6, 20.7, 20.7, 20.8, 61.5, 67.2, 71.5, 74.9, 76.7, 79.1, 169.7, 169.9, 170.0, 170.9 ppm; MS (ESI) calcd for C_14_H_20_O_9_S *m*/*z* [M + Na]^+^: 387.37, found 387.1.

**Figure 2:**
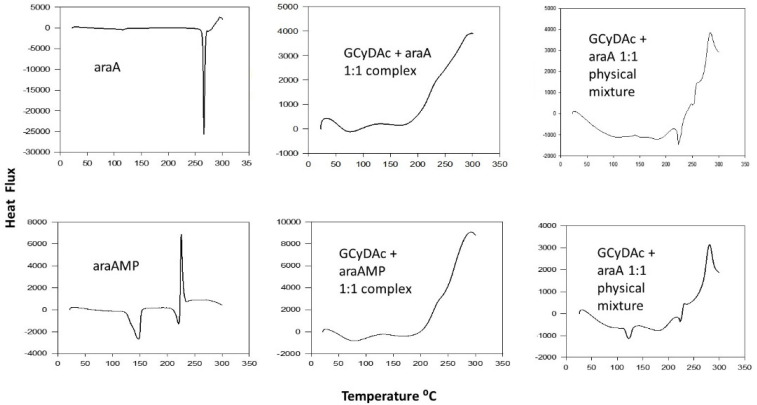
DSC thermograms of araA (upper left) and araAMP (lower left) are characterized by a sharp endothermic peak and a sharp exothermic peak, respectively. These features remain visible for the 1:1 GCyDAc:araA and GCyDAc:araAMP physical mixtures (upper right and lower right, respectively), but are essentially absent for 1:1 complexes with GCyDAc of araA and araAMP (upper and lower center frames, respectively).

*Formulation*: Test nucleosides and nucleotides were formulated with GCyDAc **8** in 1:1 molar ratios except for p-araAMP, which was formulated in a 2:1 = **8**:p-araAMP molar ratio. The complexes were prepared by the solvent evaporation method, first dissolving **8** in distilled water, then adding the test agent, sonicating (1 min), filtering (0.22 μ), and finally freeze drying. Weighed amounts (3~5 mg) of samples were sealed in aluminum pans for DSC analysis using a Seiko DSC 120 instrument. DSC scans were performed over a 20° to 300 °C range at a heating rate of 10 °C/min. Representative thermograms are presented in Figure 2.

*Cell toxicity and antiviral activity of GCyDAc formulations:* Cytotoxicity was estimated using the MTS assay (Promega North America, Madison, WI, USA) of metabolic activity in four mammalian cell lines grown in cell culture: EMT-6 (murine mammary carcinoma), KBALB (murine, virus-transformed embryonic Kirsten sarcoma), 143B (human osteosarcoma), and HepG2 (human hepatoma). Antiviral studies were performed under contract by the Katholieke Universiteit Leuven, Rega Institute for Medical Research, Belgium, using a procedure developed by Ladner et al. [33,34]. Briefly, growth of a tetracycline-responsive HepAD38 cell line, stably transfected with a cDNA copy of the pregenomic RNA of wild-type HBV virus, was initiated by tetracycline withdrawal, with or without test substance in the culture medium. The antiviral effect was quantified by measuring levels of viral DNA at day 4 post-induction, by a real-time quantitative PCR (Q-PCR), and analyzed using an SDS 7000 (Applied Biosystems, Foster City, CA, USA). A plasmid containing the full-length insert of the HBV genome was used to prepare a standard curve, and the amount of viral DNA produced in treated cultures was expressed as a percentage of the mock-treated samples.

*Molecular Docking:* Ligand preparation: the ligand, GCyDAc, was constructed using the 3D model kit of Spartan16 Parallel Suit [37,38] and preliminarily minimized using the program’s comprehensive molecular mechanics tool. Geometry optimization and energy minimization was then performed using the intuitive graphical user interface (GUI) of Spartan 16 Parallel Suit. Protein: the x-ray crystallographic structure of α-lactose complexed with ASGPr, 5jpv.pdb, was used to construct a 3D computational model for binding study [39]. Docking: both ligand and protein files required for the binding study were prepared and followed by molecular docking using AutoDock Vina [40,41].

## 3. Results

Heptakis[6-amino-6-deoxy-2-O-(3-(1′-thio-ß-D-galactopyranosyl)propyl)]-β-cyclodextrin acetate **8** (GCyDAc) was synthesized from heptakis(6-azido-6-deoxy)-β-cyclodextrin **3** in five steps, with an overall yield of 20%. Reactions were not optimized for yield.

**Figure 3:**
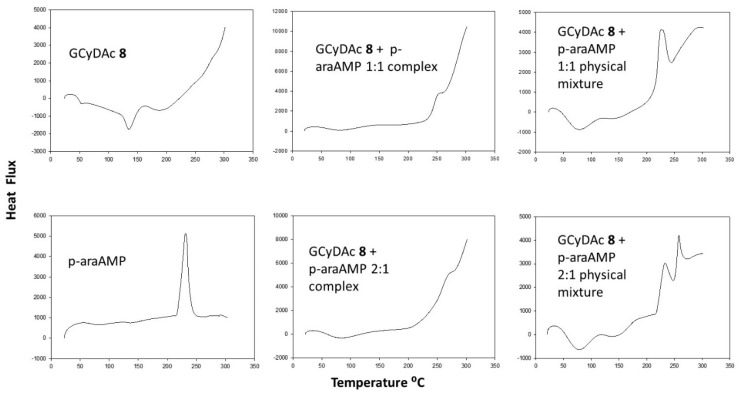
DSC thermograms of p-araAMP (lower left) and GCyDAc (upper left) alone, as complexed (center frames) and as physical mixtures (left frames). The strong exothermic peak of p-araAMP (lower left) is evident in the physical mixtures at both GCyDAc concentration ratios (1:1 upper right and 2:1 lower right), whereas it is suppressed by complexation at both ratios (1:1 upper center and 2:1 lower center). The thermal fluctuations at lower temperatures seen with GCyDAc alone (upper left) are evident in the physical mixtures (upper right and upper left) but are not present in the complexes (upper and lower center).

Differential scanning calorimetry (DSC) studies were performed on araA, araAMP, and p-araAMP, alone, as physical mixtures and as formulated complexes with GCyDAc. Thermograms shown in Figure 2 and Figure 3 depict substantial differences among the thermographs of **8**, araA, araAMP, and p-araAMP, and among thermographs of their physical mixtures and complexes with **8**.

GCyDAc was complexed with p-araAMP in both 1:1 and 2:1 ratios. The respective thermographs (Figure 3) show diminution of the nucleotide’s exothermal transition as the GCyDAc:p-araAMP ratio increased from 1:1 to 2:1.

**Figure 4:**
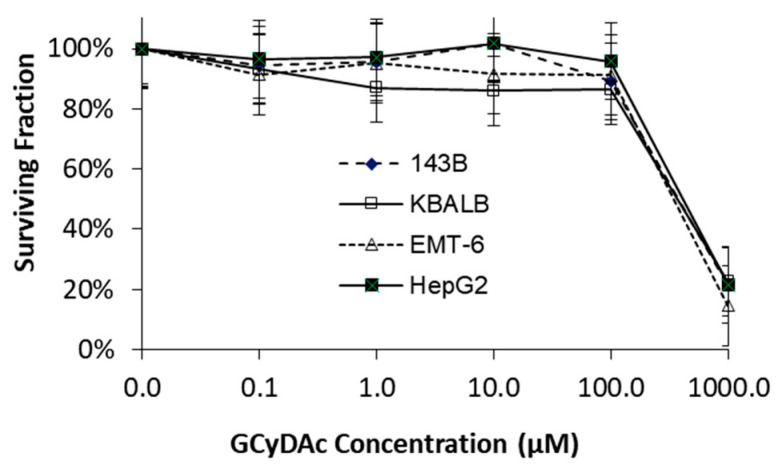
Surviving fractions of mammalian cell lines, as determined by MTT assay and expressed as a function of GCyDAc concentration.

GCyDAc was tested for toxicity against four mammalian cell lines, using a colorimetric antiproliferation assay. There was no evidence of toxicity towards these cell lines in culture, with an IC_50_ of approximately 0.5 millimolar for each cell line (Figure 4).

The antiviral activities of araA, araAMP, p-araAMP, and their respective GCyDAc formulations, are presented in Table 1. Data are expressed in molar-equivalent potencies; lamivudine (LV; 3TC; (-)-L-2′,3′-dideoxy-3′-thiacytidine) and PMPA (tenofovir; (9-[9(R)-2-(phosphonomethoxy)propyl]adenine) were used as positive controls for the antiviral assay. These data show that araA, araAMP, and p-araAMP had similar potencies, and that GCyDAc effected only minimal increases in antiviral potency. GCyDAc slightly decreased the potency of LV (reference antiviral nucleoside).

**Figure 5:**
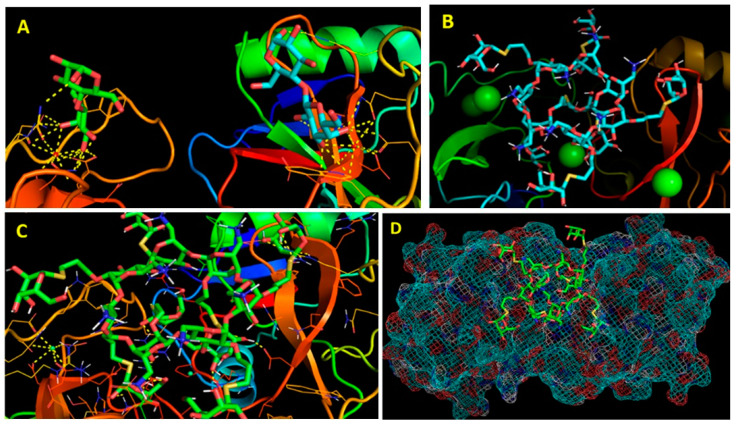
(**A**) shows the molecular interaction between lactose and ASGPr H1 domain (5jpv.pdb; [42], showing involvement of calcium ions (red dots). (**B**,**C**) show the mode of molecular binding of GCyDAc to ASGPr H1 domain (5jpv.pdb; [42]), showing involvement of calcium ions (green spheres). (**D**) shows a top view of the modeled binding of GCyDAc to ASGPr H1 domain, 5jpv.pdb.

In silico docking calculations using AutoDock Vina demonstrated the possible interaction of two galactose units of the ligand GCyDAc with the two sugar binding sites of ASGPr H1 domain (Figure 5B,C). This modeled binding is similar to the molecular interaction of two α-lactose molecules with ASGPr H1 domain (Figure 5A, 5jpv.pdb, [42]). The shallow sugar-binding pockets showed interaction, requiring calcium ions, with both hydroxyls of C-3 and C-4 of β-D-galactose. Moreover, the remaining galactose units of GCyDAc showed additional binding except for one unit, which was oriented away from the receptor-binding site, Figure 5D.

## 4. Discussion

Interest in the synthesis of substituted cyclodextrins for drug delivery remains strong after more than five decades of research [24]. The rigid 3D molecular structure of CyDs is particularly attractive for chemical elaboration because it offers two geometric surfaces for independent stereoselective derivatization [43] to precisely accommodate multiple enhancements including targeting ligands and therapeutic motifs. The immediate benefit of this spatial arrangement is augmented by the chemical reactivity of hydroxyl substituents on the two faces, with the one face projecting primary, more nucleophilic, hydroxyl groups at C-6, and the other face projecting less reactive, more acidic, secondary hydroxyl groups at C-2 and C-3, all of which can be exploited via their respective differences in chemical reactivity [44,45,46,47]. This approach has been utilized for targeted co-delivery of a range of therapeutic moieties including lectin-binding galactose motifs and therapeutic molecules such as SiRNA [48,49,50]. However, despite the promising characteristics of these delivery systems, their application in commercial products remains disappointing.

The current report addresses the exploitation of two very different functions, one targeting cellular membrane ligands, and the second based on charge-associated complexation involving alkyl amines at C-6 [30]. The influence of electrostatic interactions between host-guest, and their impact on classical bonding drivers including hydrophobic and van der Waals forces, and hydrogen-bonding, are complex and currently unpredictable. However, they have been recognized for their contributions to charge-association bonding [51]. Positively charged guanidino- and alkylamine-derivatized cyclodextrins [52] have been shown to strongly bind nucleotides through both cavity inclusion and ionic interactions in which the deoxyribose moiety, driven by phosphate-guanidino group interactions, lodges inside the cavity of per(6-guanidino-6-deoxy)-β CyD and then readily enters cancer cells to deliver monophosphorylated gemcitabine [52,53]. Overall, however, the development of charged CyD carriers has lagged behind other approaches in building, understanding, and exploiting these structures. The current manuscript extends the C-6 (primary face) amine modification to include C-2 (secondary face) hydroxyls decorated with ASGPR-reactive thio-β-D-galactopyranosylpropyl substituents.

The synthesis of GCyDAc **8** was designed to avoid lengthy synthesis methods, tedious protection–deprotection steps, and indiscriminate (sledgehammer) reactions with lengthy purification procedures [45]. The first two intermediates in the synthetic sequence (**2**, **3**; both commercially available) were prepared from β-CyD by classical methods [35,36], affording the per-azido CyD **3** in 90% yield. An acetylation–deacetylation step, introduced to facilitate purification of the poorly soluble per-azido CyD **3** on silica gel, reduced the overall recovered yield to 60%. Other syntheses have been reported, including a mechanochemical (ball mill) procedure [54] and alternative quench procedures for the initial halogenation reaction [55], both of which have the advantage of easy scale-up.

Addition of the galactose component at C-2 was achieved in two steps from per-azido-CyD **3** by first introducing a linker arm **4** and then coupling with protected thiogalactose **9** to afford **5**. Other approaches to CyD glycosylation have been reported, including the synthesis of multivalent lactose or galactose-decorated CyD via heptakis-C-6 amino-CyD [56]. Although the latter approach could have been modified to meet current needs (galactosylation at C-2), it was deemed unsuitable because it afforded a mixture of tri-, tetra-, penta-, hexa- and hepta-lactose-substituted CyDs in an overall yield of 25%, which would mitigate extensive purification to obtain fully glycosylated CyD [56]. A versatile approach to prepare per-glycosylated CyDs in 30–90% yield from a range of derivatized heptakis(6-azido-6-deoxy)-CyDs via click reactions and microwave irradiation [57] could possibly be adapted to prepare compounds that could fit within the objectives of the current project.

Aminocyclodextrins have been shown to bind nucleoside monophosphates (i.e., mononucleotides) in a geometric relationship in which the phosphate protrudes through the CyD cavity to associate with the amino group, and the sugar moiety lies within the CyD cavity [31,32,58]. Adenine-based nucleotides are bound preferentially to CyD compared to nucleotides with other nucleobases [59], but the impact of heptakis-2-O-substitution on these properties is not known. In the case of p-araAMP, classical inclusion complex formation from the secondary side of GCyDAc may be difficult because per-alkyl-thiogalactosylation at C-2 creates a barrier approximately 9.8 Å deep that protects the secondary face [30]. The current docking study showed that three of the C-2 galactoses are oriented downward, which may result in blocking access to the CyD cavity from the secondary face. Conversely, C-6 amino groups are oriented upward, forming a crown of positive charges on top of the primary face which could facilitate the interaction with the phosphate groups. Charge association for araAMP by approach from the secondary face of GCyDAc may still be possible, with the phosphate moiety penetrating through the CyD cavity and protruding from the primary face, ionically stabilized by C-6 amino groups as per the Suzuki model [31]. Additionally, in the case of p-araAMP, ionic-associated stabilization could also occur, through either terminal or backbone phosphate groups associating directly with CyD primary face alkylamino substituents, without the classical host-guest geometry, and/or by penetrating the torus from the secondary side as in the Suzuki model [31]. This could coincide with intramolecular ‘wrap-around’ or intramolecular charge associations involving additional GCyDAc molecules. A preliminary high-field NMR (800 MHz 1H) titration study (GCyDAc vs. p-araAMP) revealed concentration-dependent changes consistent with the latter concept, but the exceedingly complex spectra (not shown) precluded analysis. This work was not pursued further because resolution of these spectra would have required inclusion of an isotopic marker in the GCyDAc structure to identify the resonance of at least one participating sugar moiety.

Receptor-mediated endocytosis has long been identified as a mechanism to improve the delivery of membrane-impermeable drugs including nucleotides [60], including the delivery of RNAi using a cyclodextrin-containing polymer targeted to the transferrin receptor by human transferrin [61]. Performance-limiting properties of ASPGr as a drug delivery mechanism include its capacity constraints [62], variability of expression density in cell membranes [63], turnover (on-off and binding-internalization) times [64], and endosomal escape of transported payloads [65,66,67]. The interrelationship among these parameters is not known and no data are available specifically for araA, araAMP, and p-araAMP. However, slow receptor regeneration rates alone may create plateau/saturation kinetics that could limit the delivery and efficacy of drugs by the ASGPr receptor [62]. ASGPr expression density in the HepAD38 cell line used in the current studies, at 17,000 receptors per cell [63], is substantially lower than the 76,000 per cell reported estimate for the more commonly used HepG2 hepatic cell lines [64,65].

The ASGPr transport process starts with the formation of coated pits upon trivalent contact between the substrate and the receptor. Once triggered, membrane invagination and the formation of endosomes inside the cell proceed. The classical model for endosome processing includes a recycling component to regenerate plasma membrane and a degradative sequence that destroys entrapped ligand complexes. Early endosomes have a near neutral pH (>6), but as they mature, they become more acidic, so that the pH of late endosomes may be below 5. Drug-ligand complexes are destroyed in late endosomes, either directly by hydrolases, or following fusion with lysosomes [66,67,68]. Mechanisms of drug release from endosomes are substrate specific, complex, and largely unresolved [69,70].

The current research was based on two concepts: (1) that the fully substituted cationic CyD (GCyDAc, **8**) would form reasonably stable complexes with (oppositely charged) nucleotides (i.e., araAMP and p-araAMP), and (2) that fully galactosylated CyD (**8**) would target, bind, and activate endocytosis of the ion-stabilized complex (GCyDAc:araAMP or GCyDAC:p-araAMP). Release of the mononucleotide araAMP from the endosome would increase the antiviral effectiveness of the dose by by-passing the rate-limiting activation of the nucleoside araA. The current molecular docking model depicts only two strong binding sites between GCyDAc and ASGPr, and although other weak/cross molecular associations are possible, the inclusion of seven ‘binding sugars’ on the secondary face of GCyDAc may mitigate against full receptor activation seen with ternary binding.

Successful complexation between GCyDAc and either araA, araAMP, or p-araAMP was demonstrated by DSC. In each case (Figure 2 and Figure 3), the respective thermographs depict clear differences among the carrier, the drug, and the various physical mixtures and the complexes of the drug and carrier. The primary endothermic or exothermic transitions characteristic of thermographs of pure test substances and GCyDAc:test-substance-mixtures are absent from, or very faint in, the thermograms of the complexes. It has been shown previously by HPLC that the GCyDAc complexed with hexachlorofluorescein-tagged p-araAMP (HEX-p-araAMP) was stable in certain buffer solutions and in water, and that the 1:1 formulation contained ~90% free HEX-p-araAMP, whereas the 2:1 formulation contained <25% free HEX-p-araAMP [30]. As with other charged CyD:phosphate ester complexes [71], hydrolysis GCyDAc:HEX-p-araAMP varied by buffer, with complete dissociation within a 4 h incubation in borate buffer. Guthrie [30] further reported that their analytical method (capillary electrophoresis and laser-induced fluorescence) did not detect any complex formation between HEX-p-araAMP and the neutral CyD analog (**6**), which underscores the importance of ionic association in the complexation of p-araAMP. However, the HEX-p-araAMP findings may not translate into similar effects for p-araAMP, given the presence of the bulky (MW 741), hydrophobic HEX fluorophore that may substantially alter the association with GCyDAc, despite attachment of HEX at the terminal adenine-NH_2_.

Interaction of GCyDAc with membrane ASPGr and subsequent internalization via membrane invagination and endosome formation is a core premise of the current research. The interaction of small-molecule terminal galactose moieties with ASPGr is established in the literature, but research with sugar-derivatized CyDs and small-molecule drugs does not always support this as a major/effective drug delivery mechanism. For example, 5′-O-lactosyl-araA reached higher concentrations than araA in liver of mice following IV injection or in hepatocytes in cell culture [72], but arabinosylated-2-nitroimidazole exhibited higher in vivo uptake in liver than its corresponding galactosyl compound [73]. That is, the expected outcomes were met by lactosyl-araA but not by the galactosyl-nitroimidazole. In the case of lactosylated or galactosylated β-CyD carriers, elevated drug delivery across the blood–brain barrier and elevated hepatic drug uptake has been observed, and although endocytosis was the rationalized mechanism, the sugar density on the membrane ligand was not specified [74].

A number of plausible metabolic events may have prevented the GCyDAc complexes from providing higher antiviral activity compared to the respective non-complexed antivirals (Table 1). Plateau/saturation kinetics and slow receptor regeneration rates [64,65] could have limited the delivery of the antivirals via the ASGPr, thereby preventing cytostatic drug concentrations from being reached in HepAD38 cells. It is also possible that low antiviral activity could be due to the failure of the GCyDAc complexes to initiate internalization of the receptor. A third scenario is that even if ASPGr internalization and endosome formation did occur, araAMP was degraded prior to release into the cytoplasm. For example, endosomal nucleases may have hydrolyzed p-araAMP to the 3‘-O- rather than the 5′-O-monophosphates. This would prevent up-phosphorylation to the trinucleotide, since the 3′-O monophosphate may not be readily converted to active trinucleotide 5′-O-araATP. Production of the 3′-O- vs. the 5′-O-monophosphates is known to be nuclease-dependent and substrate (polynucleotide)-dependent; for example, DNA and RNA are hydrolyzed to deoxyribomonophosphates and ribo-3′-monophosphates, respectively [75,76]. To our knowledge, the products of chemical hydrolysis or enzymatic hydrolysis products of poly-arabinosyl nucleotides are not reported. It is also possible that degradation processes other than hydrolysis effected inactivation of endosomal GCyDAc:p-araAMP. Deaminases, for example, could limit antiviral activity of GCyDAc-complexed araAMP and p-araAMP via metabolic deamination to their corresponding, inactive, inosine metabolites, which would parallel a major metabolic pathway active in the degradation of adenosine and its mononucleotide [77,78].

## 5. Summary

The objectives of the current research were to develop a receptor-targeted delivery agent to by-pass the rate-limiting cellular activation (5′-monophosphorylation) of antiviral/anticancer nucleosides (i.e., araA) and to simultaneously improve the delivery of their respective mononucleotides (i.e., araAMP) and polynucleotides (p-araAMP). AraA was selected for this demonstration because, like many antiviral nucleosides, it requires three-step anabolic phosphorylation to its triphosphate (i.e., araATP) in order to effect inhibition of viral DNA synthesis, its main mechanism of antiviral action. Since the first step, monophosphorylation, is rate-limiting, delivery of araAMP as a drug or as a metabolite of p-araAMP could effectively by-pass rate-limiting monophosphorylation.

GCyDAc was chosen as the nucleotide carrier, offering seven primary-side charge associations per carrier molecule, and at the same time, targeting the ASGPr ligand on hepatocyte membranes via its seven secondary side galactose arms. The ASGPr targeting/transport system was selected to deliver the nucleotides via its endosome-forming mechanism, with the goal of delivering the nucleotides and releasing activated drug intermediates (e.g., araAMP) into the hepatocyte. The efficacy of this process would enhance viral antiviral potency as determined by cell cytotoxicity in the HepAD38 cell line in cell culture.

It has been stated that CyD drug delivery research today needs novel cyclodextrins with demonstrated therapeutic effectiveness in addition to those currently approved [79]. The current report describes a high-density charge-association binding mechanism that could potentially supplement fundamental CyD inclusion complexation forces, and simultaneously targets cell membrane ligands to initiate payload importation via endosome formation. This approach supplements contemporary understanding of cyclodextrin-based drug delivery [80].

## Figures and Tables

**Table 1:** Antiviral potencies (EC_50_) of araA, araAMP, p-araAMP, two active anti-HBV antivirals, and their GCyDAc formulations, against hepatoma HepAD38 cells in cell culture.

Compound/Complex	Test Conc. (µg/mL)	EC_50_ (µg/mL) (araA Equivalent)	Potency Relative to araA
GcyDAc (ASGPr targeted carrier)	0.8–100	>100	N/A
Lamivudine * (LV,3TC) (reference antiviral)	0.8–100	<0.8	43.7
GcyDAc:LV (reference antiviral complex)	0.8–100	1.5	23.3
PMPA ** (reference antiviral)	0.08–10	0.7	50
araA	0.8–100	35	1.0
GcyDAc:araA (1:1)	0.8–100	14	2.5
araAMP	0.8–100	33	1.1
GcyDAc:araAMP (1:1)	0.8–100	26	1.3
p-araAMP	0.8–100	29	1.2
GcyDA:p-araAMP (2:1)	0.8–100	29	1.2

* Lamivudine (LV); 3TC; (−)-L-2′,3′-dideoxy-3′-thiacytidine; ** PMPA (Tenofovir); (9-[9^®^-2-(phosphofind and changenomethoxy)propyl]adenine).

## Data Availability

Requests for data access should be addressed to the corresponding author.

## Supplementary Materials

The following supporting information can be downloaded at: https://www.mdpi.com/article/10.3390/pharmaceutics16030323/s1, Figure S1: p-araAMP gels and CE.

## Abbreviations

AMP (adenosine-5′-monophosphate); araA (9-arabinosyl adenine, 9-β-D-arabinofuranosyl adenine; vidarabine); araAMP (arabinosyl adenine-5′-monophosphate); ASGPr (asialoglycoprotein receptor); CyD (cyclodextrin); DSC (differential scanning calorimetry); GCyDAc (**8**; heptakis[6-amino-6-deoxy-2-O-(3-(1′-thio-β-D-galactopyranosyl)propyl)]-β-cyclodextrin acetate); OAc-TG (**9**; 2,3,4,6-tetra-O-acetyl-1-thio-β-D-galacto-pyranose); p-araAMP [12-*mer*-polyaraAMP; 5′-phosphodeoxyadenlyl(5′-3′)(araAMP)_11_].
