# Supplementary material for: Synthesis and Preliminary Evaluation of an ASGPr-Targeted Polycationic β-Cyclodextrin Carrier for Nucleosides and Nucleotides
Source: Pharmaceutics. 2024 Feb 26;16(3):323. doi: 10.3390/pharmaceutics16030323 (PMC10975686; doi:10.3390/pharmaceutics16030323)
Supplement: Supplementary file 1 [file pharmaceutics-16-00323-s001.zip › pharmaceutics-2852473-supplementary.pdf]

pharmaceutics

Supplementary Material

Synthesis and Preliminary Evaluation of an ASGPr-targeted Polycationic β-cyclodextrin Carrier for Nucleosides and Nucleotides

Jang-Ha (John) Ryu, [1], Weizhong Zheng, [2], Xiao-Hong Yang, [3], Hassan Elsaidi, [4], Jim Diakur, [5†], Leonard I. Wiebe [*†]

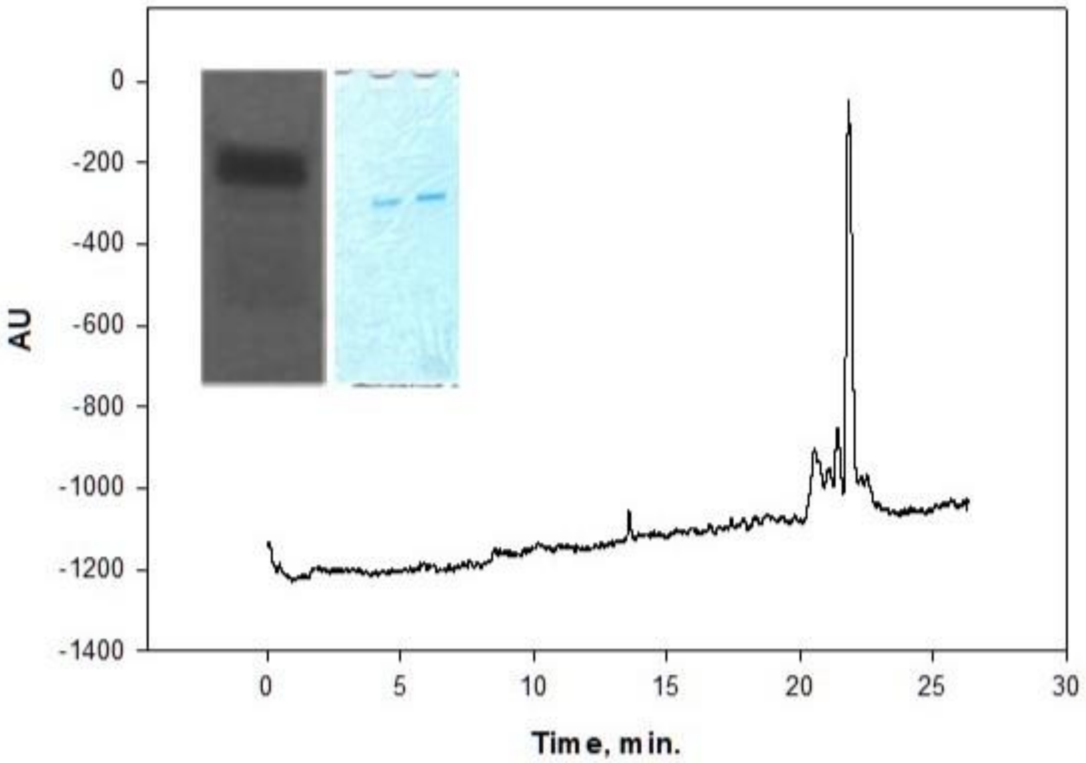

Figure S1. p-araAMP gels & CE.
